# Evolving Therapeutic Algorithms in Chronic Myeloid Leukemia: Integrating Efficacy, Safety, and Survivorship

**DOI:** 10.3390/biomedicines14020408

**Published:** 2026-02-11

**Authors:** Yan Leyfman, Ahmed Hashim Azeez, Taha Kassim Dohadwala, Soumiya Nadar, Riya Vaishnav, Sumaiya Khan, Vraj JigarKumar Rangrej, Viviana Cortiana, Chandler Park

**Affiliations:** 1 NewYork Presbyterian Hospital, Brooklyn, NY 11215, USA; yan.leyfman@nyp.org; 2 Department of Medicine, Tbilisi State Medical University, Tbilisi 0186, Georgia; ahmedhashim22@gmail.com (A.H.A.); soumiya.n2001@gmail.com (S.N.); 3 Department of Medicine, David Tvildiani Medical University, Tbilisi 0159, Georgia; 4 Department of Medicine, Medical College Baroda, Vadodara 390001, Gujrat, India; riya.v9916@gmail.com; 5 Department of Medicine, Yerevan State Medical University, Yerevan 0025, Armenia; duax10k@gmail.com; 6 Department of Medicine, GMERS Medical College Gotri, Vadodara 390021, Gujrat, India; rangrejvraj13@gmail.com; 7 Department of Medical and Surgical Sciences (DIMEC), University of Bologna, 40126 Bologna, Italy; viviana.cortiana@studio.unibo.it; 8 Norton Cancer Institute, Louisville, KY 40202, USA; chandler.park@louisville.edu

**Keywords:** chronic myeloid leukemia, tyrosine kinase inhibitors, molecular monitoring, deep molecular response, treatment-free remission

## Abstract

Chronic myeloid leukemia (CML) has undergone a significant shift over the past two decades, transitioning from a fatal malignancy to a chronic, highly manageable disease with near-normal life expectancy for most patients. This transformation has been driven by the development of BCR-ABL1-targeted tyrosine kinase inhibitors (TKIs), which have enabled durable disease control and deep molecular responses (DMRs) in the majority of patients with chronic-phase CML. As long-term survival outcomes have plateaued across available agents, contemporary management has shifted beyond disease suppression toward optimizing long-term safety, quality of life, and the achievement of treatment-free remission (TFR). This review summarizes current evidence on molecular monitoring strategies, the comparative efficacy and toxicity profiles of first-, second-, and third-generation TKIs, and emerging advances in response assessment. Patient-centered TKI selection is discussed in the context of cardiovascular risk, comorbidities, treatment tolerability, and survivorship goals, reflecting the growing emphasis on individualized therapy in chronic-phase CML. Molecular monitoring strategies are examined in parallel, highlighting the clinical importance of early and sustained DMRs in guiding therapeutic decisions and TFR eligibility. Although RT-qPCR remains the standard for molecular monitoring, emerging high-sensitivity techniques such as digital droplet PCR and next-generation sequencing provide complementary value by improving the detection of low-level residual disease, refining risk stratification, and enabling earlier identification of resistance. Emerging therapeutic strategies and advances in response assessment further highlight ongoing efforts to enhance the depth and durability of remission while minimizing long-term toxicity. These developments support a more precise, individualized, and outcome-driven approach to modern CML management.

## 1. Introduction

Chronic myeloid leukemia (CML) is a clonal myeloproliferative neoplasm with a global incidence ranging from 0.4 to 1.75 cases per 100,000 individuals annually, accounting for approximately 15% of all adult leukemias [1,2]. In 2021, approximately 35,830 new cases were reported globally, with a male predominance (male-to-female ratio: 1.2–1.7) and a median age at diagnosis of 65–66 years [1,3,4]. However, this varies regionally, with younger medians reported in low-income countries [2,5,6].

The biological foundation of CML is the Philadelphia (Ph) chromosome, a reciprocal translocation t(9;22)(q34;q11.2) which fuses the BCR gene on chromosome 22 with the ABL1 proto-oncogene on chromosome 9 [7,8]. This fusion generates the BCR-ABL1 oncoprotein, a constitutively active tyrosine kinase that dysregulates signaling pathways, promoting uncontrolled proliferation, resistance to apoptosis, and genomic instability in hematopoietic stem cells [7,9,10].

Historically, therapeutic strategies for CML were limited to busulfan, hydroxyurea, interferon-alpha (IFN-α), often with low-dose cytarabine, or allogeneic stem cell transplantation (allo-HSCT) [11,12,13]. These modalities achieved only modest disease control, with median survivals of approximately 4–6 years and 5-year overall survival rates ranging from 20 to 50% [11,14]. The introduction of imatinib in 2001, followed by the development of second- and third-generation tyrosine kinase inhibitors (2G- and 3G-TKIs), including dasatinib, nilotinib, bosutinib, ponatinib, and asciminib, fundamentally transformed CML management by targeting the BCR-ABL1 oncoprotein [15,16]. TKIs have greatly improved outcomes, with 5-year overall survival (OS) exceeding 80% compared to <50% in the pre-TKI era [11,17]. Life expectancy now approximates that of the general population for many patients, with 8-year survival rising from 6% before 1975 to 87% post-2001 [17,18].

With the transformative impact of TKIs achieving near-normal life expectancy for most patients with chronic-phase CML, overall survival is no longer the primary therapeutic endpoint. Current goals now prioritize achieving sustained deep molecular responses (DMRs) to enable treatment-free remission (TFR) and long-term quality of life (QoL) [19,20]. However, significant challenges persist, including chronic low-grade toxicities such as fatigue, musculoskeletal pain, and gastrointestinal effects that impair health-related QoL [21,22], an increased risk of serious cardiovascular (CV) and arteriothrombotic events with specific TKIs such as nilotinib and ponatinib [23,24], suboptimal adherence associated with indefinite daily therapy [25], and economic burden due to lifelong treatment [26,27].

This review, inspired by Jorge Cortes, MD’s keynote lecture at MedNews Week [28], aims to explore therapeutic algorithms in CML, integrating efficacy, safety profiles, and survivorship considerations to guide personalized management in this era of prolonged remission.

## 2. Molecular Assessment of Treatment Response in Chronic Myeloid Leukemia

The management of CML has undergone a significant transformation, transitioning from rudimentary hematologic assessments to highly sophisticated molecular monitoring [29]. Historically, treatment success was dictated by Complete Cytogenetic Response (CCyR), characterized by the absence of the Ph chromosome in at least 20 bone marrow metaphases [30]. However, with the clinical introduction of TKIs, the depth of response reached levels where conventional cytogenetics lacked the necessary sensitivity to detect residual disease. This necessitated the adoption of Real-Time Quantitative PCR (RT-qPCR) to measure BCR-ABL1 mRNA transcript levels [31].

RT-qPCR, reported on the International Scale (IS), has become the global standard for disease monitoring because it provides a logarithmic scale of tumor burden reduction [30]. The IS expresses the ratio of BCR-ABL1 transcripts to an internal control gene (commonly ABL1, beta-2-microglobulin, and beta-glucuronidase) as a percentage, with 100% being the standardized baseline established in the IRIS (International Randomized Study of Interferon and STI571) trial [32]. The standardization of this scale through laboratory-specific conversion factors allows for the comparison of results across different global centers, which is vital for maintaining uniform care standards [33].

CCyR, defined by a BCR-ABL1 IS ≤ 1%, remains the gold standard for optimal outcomes in CML [34,35]. Achieving CCyR within the first 12 months of therapy is strongly associated with prolonged progression-free survival and a significantly reduced risk of transformation to the accelerated or blast phases [36]. Building upon this, major molecular response (MMR) is defined as BCR-ABL1 IS ≤0.1% (a 3-log reduction) [35]. MMR is widely regarded as the “safe harbor” in CML; patients who maintain an MMR have a negligible risk of disease progression, making it the primary goal of long-term TKI therapy [37].

For patients seeking TFR, achieving a DMR is mandatory [38]. DMR is categorized as a molecular response with a 4-log reduction (MR4) (≤0.01%), a molecular response with a 4.5-log reduction (MR4.5) (≤0.0032%), or a molecular response with a 5-log reduction (MR5) (≤0.001%) [39]. Current guidelines and clinical trials, such as STIM, have demonstrated that a minimum duration of TKI therapy of 3 years, sustained DMR for at least 2 years, and a molecular response of at least MR4 are criteria for TKI discontinuation [40,41].

**Figure 1:**
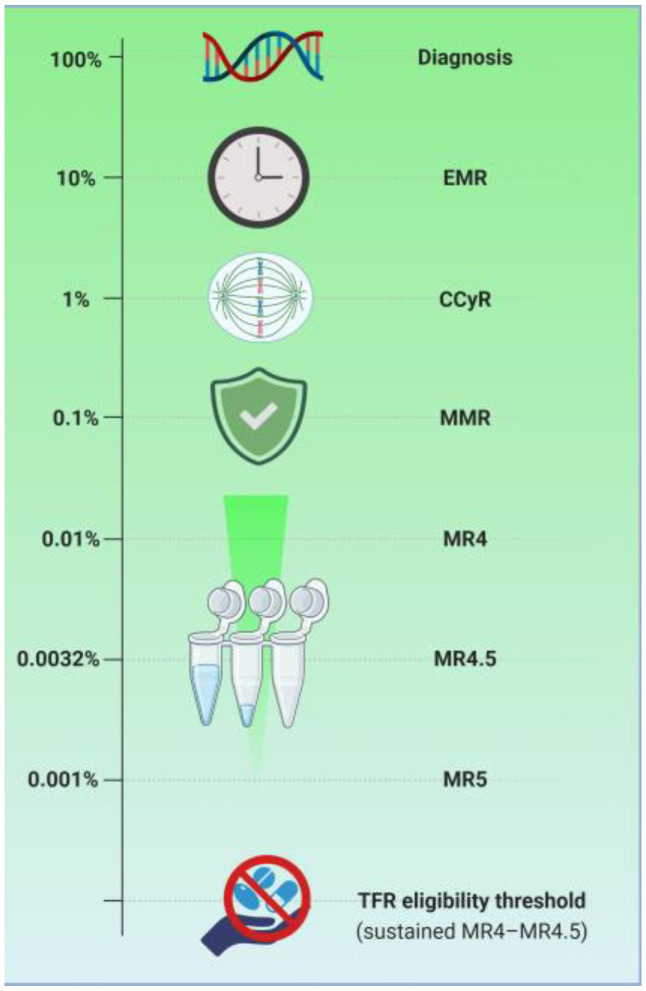
The depth of molecular response and corresponding monitoring modalities in chronic myeloid leukemia. Progressive molecular response levels according to the International Scale of BCR-ABL1 transcripts are shown: from diagnosis (100%) through CCyR (1%), MMR (≤0.1%), MR4 (≤0.01%), MR4.5 (≤0.0032%), and MR5 (≤0.001%). Sustained DMR (MR4 or MR4.5 maintained over time) represents the eligibility threshold for TFR. Created in BioRender, Dohadwala, T. (2026), at https://BioRender.com/nwix7w7 (accessed on 29 December 2025).

The kinetics of transcript reduction are evaluated at time-dependent milestones (3, 6, and 12 months) [42]. Early molecular response, defined as BCR-ABL1 IS ≤ 10% at 3 months, has emerged as a critical early predictor of long-term outcomes [43]. In the most recent European LeukemiaNet (ELN) recommendations, patients with high-risk additional chromosomal abnormalities (ACA), previously categorized as a “warning,” are now classified as high risk, reflecting their lower likelihood of achieving MMR or DMR and their inferior survival outcomes [29]. Conversely, a loss of molecular response, defined primarily as the loss of MMR or a confirmed 1-log increase in transcript levels, requires immediate clinical intervention [44]. This involves assessing patient adherence, screening for BCR-ABL1 kinase domain mutations, and potentially switching to a more potent TKI [44]. Despite the precision of PCR, bone marrow cytogenetics still plays a foundational role in diagnosis to identify ACA and remains necessary if treatment failure or disease progression is suspected [45] (Table 1) [Figure 1].

## 3. Limitations and Emerging Advances in Molecular Monitoring

While RT-qPCR remains the clinical standard for molecular monitoring in CML, it is subject to several important technical and biological limitations. Assay sensitivity is intrinsically dependent on RNA integrity and on the number of control gene transcripts quantified [46]. For instance, reliable assessment of DMR at the MR4.5 level requires the detection of at least 32,000 ABL1 transcripts; samples failing to meet this threshold are considered non-informative for evaluating deep response status [47]. In such cases, BCR-ABL1 transcripts may appear undetectable despite the persistence of residual leukemic burden, reflecting inadequate sample quality rather than true molecular eradication [48]. Inter-laboratory variability further complicates RT-qPCR interpretation. Although harmonization through the IS has improved comparability, differences in RNA extraction methods, assay platforms, calibration procedures, and analytical sensitivity, particularly at very low transcript levels, can result in discordant measurements across laboratories [49].

Beyond analytical constraints, pre-analytical and biological factors significantly influence RT-qPCR results [50,51]. Pre-analytical variables such as sample collection timing, handling, and transport conditions can significantly influence quantitative results [52]. Additionally, biological fluctuations in BCR-ABL1 transcript levels may occur independently of true changes in disease burden, particularly at very low levels of residual disease [53,54]. This issue is particularly relevant near clinical decision points for MMR, DMR, or TFR eligibility, where small numerical differences may carry disproportionate clinical consequences. Accordingly, confirmatory testing, longitudinal trend analysis rather than single time-point assessment, and the integration of clinical context are essential to avoid inappropriate treatment modification or premature therapy discontinuation.

To overcome these limitations, digital droplet PCR (ddPCR) has emerged as a promising complementary technology [55,56]. By partitioning samples into thousands of nanoliter-sized droplets, ddPCR enables absolute quantification of BCR-ABL1 transcripts, achieving greater precision and a lower limit of detection without reliance on standard curves. Growing evidence indicates that ddPCR may more accurately identify patients capable of sustaining TFR compared with conventional RT-qPCR [56,57]. In parallel, next-generation sequencing (NGS) has transformed mutation analysis by detecting resistant BCR-ABL1 clones at frequencies as low as 1%, substantially exceeding the 15–20% sensitivity of traditional Sanger sequencing [58,59]. The integration of these high-sensitivity molecular tools will be essential for refining TFR eligibility and further personalizing therapy in the era of precision hematology.

Despite the increasing availability of highly sensitive molecular techniques such as ddPCR and NGS, their use in routine practice remains limited, largely confined to clinical trials. Implementation is constrained by infrastructure requirements, a lack of standardization, and cost, which restricts access across institutions and healthcare systems [60]. In addition, further international harmonization and validation are needed before digital PCR can be widely adopted in routine diagnostics [61]. Acknowledging these limitations is important for placing molecular monitoring strategies and TFR recommendations into an appropriate clinical context.

While advances in molecular monitoring provide increasingly precise tools for disease assessment, successful CML management also relies on consistent treatment adherence. Adherence requires a shared effort from patients, healthcare providers, and the healthcare system. When patients understand the importance of consistent therapy and are supported through education, monitoring, and system-level resources, adherence improves. Challenges include complex regimens, treatment-related side effects, financial barriers, and psychosocial factors, all of which can compromise outcomes if unaddressed [25,62,63]. Improving adherence relies on patient-centered education, clear and ongoing communication, and supportive interventions such as counseling, reminders, and structured follow-up [64,65]. Together, these strategies help optimize treatment effectiveness, reduce disease progression risk, and improve QoL for patients with CML. Beyond its role in response assessment and resistance detection, molecular monitoring provides a framework for individualized therapeutic decision-making in CML, directly informing both frontline TKI selection and subsequent treatment modification.

## 4. Tyrosine Kinase Inhibitor Therapy: Comparative Efficacy and Frontline Selection

**Figure 2:**
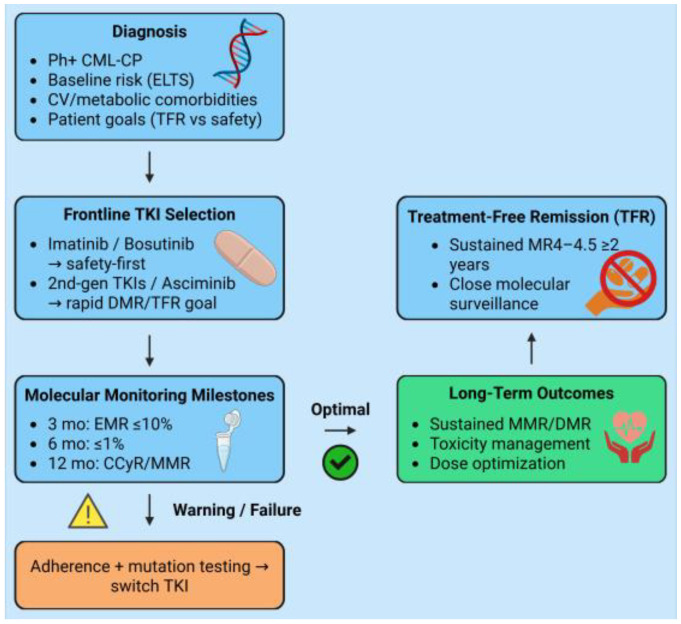
Integrated therapeutic algorithm for chronic-phase chronic myeloid leukemia (CML). Created in BioRender, Dohadwala, T. (2026), at https://BioRender.com/pbecqn5 (accessed on 29 December 2025).

TKIs have transformed chronic-phase CML from a life-threatening disease into a manageable long-term condition, with most patients now achieving a near-normal life expectancy [18,66]. Consequently, selecting an initial TKI is no longer based on disease control. Instead, it involves balancing the depth of response with long-term toxicity, cardiovascular (CV) risk, and the potential to achieve TFR (Figure 2).

### 4.1. Landmark and Emerging Trials Informing CML Management

The IRIS trial established imatinib as the foundational standard of care in chronic-phase CML, demonstrating durable CCyR and MMR and a sustained survival advantage over interferon-based therapy [67]. Subsequent randomized phase III trials, notably DASISION and ENESTnd, compared the second-generation TKIs dasatinib and nilotinib, respectively, with imatinib in the frontline setting [68,69,70,71]. These studies, together with recent meta-analyses, consistently show higher rates of early CCyR and MMR with 2G-TKIs, although without a clear OS advantage. Consequently, imatinib, dasatinib, nilotinib, and bosutinib are currently accepted frontline options for chronic-phase CML, with agent selection guided by disease risk, comorbidity profile, and toxicity considerations (Table 2).

Ponatinib, approved based on the PACE trial, provides an effective option for patients resistant to prior TKIs, including those with the T315I mutation, and later studies, such as the OPTIC trial, have refined dose-adjusted strategies to maintain efficacy while reducing the risk of arterial occlusive events [72,73]. More recent studies have shifted attention toward treatment sequencing and tolerability in later lines. The phase III ASCEMBL trial compared the allosteric STAMP inhibitor asciminib with bosutinib in patients with resistance or intolerance to at least ≥2 TKIs, demonstrating superior MMR and improved treatment continuity with asciminib [74]. The results from the second-line cohort of ASC2ESCALATE are similarly encouraging, although longer follow-up is needed to define durability, long-term tolerability, and the effectiveness of dose escalation strategies [75].

Several ongoing and recently completed trials further reinforce the translational relevance of response-driven therapy. ASC4FIRST evaluated asciminib as frontline therapy compared with the investigator’s choice of ATP-competitive TKIs, aiming to determine whether highly selective BCR-ABL1 inhibition can deliver DMRs with improved tolerability [76]. In parallel, large TFR-focused trials, including EURO-SKI, STIM, and DESTINY, have characterized eligibility criteria and relapse kinetics following TKI discontinuation, thereby directly informing current therapeutic objectives and discontinuation strategies in CML [40,77,78]. Subsequent trials, including ENESTfreedom [79] and ENSTop [80], demonstrated the feasibility of TFR following nilotinib-based therapy, while DASFREE [81] and DADI [82] extended these observations to dasatinib-treated populations. More recent investigations, such as DANTE (NCT03874858) [83] and DAstop2 (NCT03573596) [84], are evaluating second TFR attempts following retreatment or consolidation strategies, addressing an important unmet need for patients who relapse after an initial discontinuation attempt.

These studies highlight that achieving early and sustained DMR is associated with improved long-term disease control and a higher probability of successful TFR. At the same time, careful selection of therapeutic agents and optimized dosing strategies can minimize overall toxicity.

### 4.2. Patient-Adapted Frontline and Early-Line Selection

In contemporary practice, the choice of frontline TKI in CML is increasingly individualized. 2G-TKIs may be preferred in younger patients with a strong intent to pursue TFR, given their ability to achieve rapid and sustained DMR. Likewise, individuals with high ELTS risk scores may benefit from a more potent agent upfront. Conversely, comorbidities such as pulmonary disease, diabetes, or CV risk may guide avoidance of specific TKIs, highlighting the need for a tailored approach to therapy [30,85,86]. However, imatinib continues to represent a suitable frontline option, particularly in patients with low-risk disease or those with significant comorbidities, given its well-established long-term safety profile, durable efficacy, and cost-effectiveness compared with 2G-TKIs [86].

Prognostic scores such as Sokal and Hasford were developed in the chemotherapy and interferon eras and have limited applicability in contemporary CML management, often overestimating risk, particularly in older patients, under TKI therapy [87,88,89]. While the ELTS score better predicts CML-related mortality in the TKI era, baseline prognostic scores alone do not take into account important factors such as comorbidities and dynamic treatment responses, and therefore may be insufficient for fully guiding individualized clinical decisions [90,91]. Current guidelines, therefore, emphasize dynamic, response-based assessment using early molecular milestones, alongside individualized assessment of comorbidities and treatment tolerability, as the primary guide for therapy decisions [30].

### 4.3. Emerging Agents and Future Directions

Beyond currently approved TKIs, several next-generation BCR-ABL1 TKIs are under active clinical development, aiming to address resistance, intolerance, and long-term safety limitations associated with earlier agents. Olverembatinib (HQP1351), a potent 3G-TKI with activity against the T315I mutation, has demonstrated clinically meaningful responses in heavily pretreated patients, including those with multi-TKI resistance, and is being evaluated in resistant chronic- and advanced-phase CML [92]. Vodobatinib (K0706) has also shown activity across multiple BCR-ABL1 resistance mutations, with early clinical data suggesting a favorable balance between molecular efficacy and tolerability in patients resistant or intolerant to prior TKIs [93]. In parallel, early-phase agents such as ELVN-001 and TGRX-678 are currently being evaluated in preliminary trials to overcome resistance mechanisms and improve long-term tolerability [94,95].

BCR-ABL1-targeted proteolysis-targeting chimeras induce selective degradation of the fusion oncoprotein via ubiquitin–proteasome pathways, representing a novel strategy to eliminate CML cells resistant to conventional TKIs [9,96,97]. Complementary immunotherapeutic approaches, including chimeric antigen receptor T cells, bispecific T-cell engagers, and antigen-specific vaccines, aim to eradicate residual leukemic stem cells (LSCs) and enhance deep molecular responses [98,99,100]. Combination regimens integrating TKIs with LSC-targeting or immune-based therapies represent a particularly promising option to achieve durable remissions.

The integration of these emerging therapies into clinical practice will depend not only on molecular efficacy but also on long-term safety, tolerability, and their ability to facilitate sustained TFR. Biomarker-driven trials and precision medicine approaches will be essential to match patients with the optimal therapy, taking into account mutation profiles, comorbidities, and personal treatment goals. As multiple agents progress through early and late-phase clinical development, the next decade will potentially allow for a more personalized, biology-driven approach to CML management, with the potential to transform long-term outcomes and QoL.

## 5. Long-Term Safety, Tolerability, and Quality-of-Life Outcomes

Long-term exposure to TKIs is associated with a well-characterized spectrum of chronic toxicities. These include hematologic adverse effects, most commonly neutropenia and thrombocytopenia, as well as a range of non-hematologic complications such as persistent fatigue, gastrointestinal disturbances, and cutaneous reactions. In addition, prolonged TKI therapy has been linked to CV and metabolic toxicities, fluid retention syndromes, and low-grade hepatic and pancreatic enzyme elevations [101,102,103]. While all TKIs share overlapping adverse effect profiles, the frequency and type of toxicities vary among agents. These differences in chronic toxicity profiles are clinically relevant and often influence initial TKI selection and long-term management, particularly in patients with pre-existing comorbidities.

Fatigue is the most common and troublesome symptom reported by patients receiving dasatinib, imatinib, nilotinib, and ponatinib [21,104]. Gastrointestinal toxicity, particularly diarrhea, is most prominent with bosutinib, as highlighted across various studies [21,105,106,107]. Symptoms such as fatigue, muscle cramps, and dermatologic complaints are typically low-grade and subjective, contributing to underreporting and frequent underestimation by clinicians, even during long-term follow-up with the same physician [108]. This shows a clear gap between standard clinical assessment and the patient’s actual symptom burden, stressing the need for a more proactive and systematic approach to symptom monitoring and management.

Cardiovascular disease (CVD) and CV risk factors are highly prevalent in patients with CML and contribute substantially to morbidity and mortality in this population [109]. At diagnosis, approximately 18% of patients already have established CVD, and nearly 44% fall into an intermediate-to-high risk category for future CV events [109,110]. Current evidence indicates that CV AEs associated with TKI therapy occur predominantly in patients with pre-existing CV risk factors [24,111]. CV AEs are reported with greater frequency in patients treated with 2G-TKIs, including nilotinib and dasatinib, as well as the 3G-TKI ponatinib, all of which have been associated with an increased incidence of vascular occlusive and ischemic complications relative to imatinib [112]. Frequently reported clinically significant CV toxicities among the newer generation of TKIs include myocardial infarction, stroke, peripheral arterial disease, QT prolongation, pleural effusions, and systemic or pulmonary hypertension [111,112,113]. To minimize these risks, preventive care should focus on aggressive baseline CV risk assessment, optimization of modifiable risk factors, and vigilant monitoring. Individualized treatment and continuous follow-up are essential to ensure both efficacy and safety [114].

2G-TKIs are associated with higher rates of DMR than imatinib and are therefore preferentially selected with the explicit objective of pursuing TFR [86,115,116]. However, long-term tolerability plays a critical role in determining whether these molecular advantages translate into sustained, uninterrupted therapy. Across discontinuation studies of dasatinib and nilotinib, TFR rates of approximately 40 to 60% have been reported among highly selected patients with sustained DMR, supporting the feasibility and safety of treatment discontinuation in this setting [79,117,118,119,120,121]. In a large Italian real-world cohort, patients who discontinued 2G-TKIs demonstrated numerically higher adjusted TFR rates than those stopping imatinib, and multivariable analysis identified prior 2G-TKI exposure as independently associated with improved TFR outcomes [122].

Long-term follow-up analyses of 1L-2G-TKI therapy in chronic-phase CML indicate that over 40% of patients eventually require treatment switching, most frequently due to intolerance rather than resistance, with approximately 26% discontinuing for intolerance alone [123]. Similar treatment switch or discontinuation rates of 35–40% have been observed in the pivotal DASISION and ENESTnd trials, highlighting that intolerance remains the predominant driver of therapy modification despite the efficacy of 2G-TKIs [123,124,125]. Consequently, while 2G-TKIs enhance the depth and rapidity of molecular responses and increase the proportion of patients who qualify for TFR, their less favorable long-term tolerability profiles lead to higher rates of intolerance-driven discontinuation or switching. This limits the proportion of the overall treated population able to sustain the prolonged, uninterrupted DMR required for successful TFR.

Although patients with CML now have near-normal life expectancy, persistent low-grade adverse events, along with the financial pressure of chronic therapy, can affect their QoL [18,126,127]. Patient-reported outcomes (PROs) offer a direct measure of symptom burden and QoL, supporting a more patient-centered approach to care. As such, PROs are an essential component of CML management [128,129]. Clinical trials, including ASC4FIRST, have demonstrated the feasibility of integrating PROs into the evaluation of therapeutic efficacy [130]. Complementary real-world data indicate that systematic symptom monitoring can enhance treatment adherence, facilitate early molecular responses, and improve patient–physician communication [131]. These findings underscore the utility of PROs in guiding therapeutic decision-making, particularly given the established correlation between early molecular response, long-term survival, and eligibility for TFR.

In elderly or high-risk patients, long-term TKI therapy can lead to high rates of treatment failure and non-CML-related deaths. This demands dose optimization strategies such as dose reduction, de-escalation, or intermittent dosing [132,133,134]. The goals of dose optimization are to maintain efficacy, minimize adverse events, improve QoL, and support TFR. Initial TKI dosing is standard dosing and may be tailored to individual patient characteristics [133,135]. Retrospective studies indicate that TKI dose reduction before discontinuation is feasible and may preserve TFR, although prospective data are needed to confirm long-term outcomes. Such strategies are particularly pertinent in older patients, where cumulative toxicity is a concern [136,137,138].

Apart from clinical outcomes and dosing considerations, long-term TKI therapy also carries significant psychosocial impacts for patients living with CML [126,139]. A recent nationwide survey reports that patient priorities vary with age. Younger patients often worry more about fertility, sexual health, and staying active at work and socially. In comparison, older patients tend to focus on mental well-being, independence in daily life, and treatment tolerability [140]. During the COVID-19 pandemic, patients with CML were found to have a higher prevalence of depression, anxiety, and psychological distress compared with non-cancer controls, which may adversely affect treatment adherence and clinical outcomes [141]. Prolonged therapy can also impose significant financial strain, further impacting daily functioning. Recent evidence indicates that, beyond the symptomatic burden, treatment modifications are associated with increased healthcare resource utilization and higher costs [142]. These findings highlight the importance of systematically addressing both the psychosocial and economic consequences of CML management as integral components of long-term patient care.

## 6. Resistance Mechanisms and Molecular Heterogeneity in Chronic Myeloid Leukemia

Resistance to TKIs in BCR-ABL1-positive leukemias is primarily driven by alterations in the BCR-ABL1 kinase domain (KD), where point mutations impede inhibitor binding while preserving the kinase’s oncogenic activity [8,143]. BCR-ABL1 KD mutations, along with genomic amplification, represent the best characterized mechanisms of resistance in CML. Identification of these mutations in TKI-treated patients may indicate impending disease progression, as chronic-phase leukemic cells harbor gene expression patterns reminiscent of blast crisis [144]. Despite high initial response rates to BCR-ABL1-targeted TKIs, both primary and secondary resistance can develop, resulting in suboptimal outcomes and increased risk of progression [145,146].

Point mutations within the BCR-ABL1 KD represent the predominant mechanism of TKI resistance in CML, with over 100 distinct substitutions reported that cluster in functional regions such as the phosphate-binding loop, catalytic domain, activation loop, and imatinib-binding site. Common clinically significant substitutions include G250E, Y253F/H, E255K/V, T315I, M351T, and F359V, each capable of altering TKI binding or kinase conformation [8,147]. The Thr315 residue serves as a critical gatekeeper at the ATP-binding pocket, and the T315I substitution not only disrupts essential hydrogen bonding with TKIs but also creates steric hindrance that renders most ATP-competitive inhibitors ineffective [148,149].

Given this therapeutic challenge, ponatinib and asciminib currently represent the principal targeted treatment options for patients with BCR-ABL1-positive leukemias harboring the T315I mutation, addressing a historically high-risk molecular subgroup with limited sensitivity to earlier-generation TKIs [72,150]. Although asciminib exhibits activity against BCR-ABL1 T315I, its efficacy is not uniform across all T315I-positive cases. Clinical responses may be reduced in the presence of additional or compound KD mutations, which can impair allosteric binding or promote alternative resistance mechanisms [151,152,153]. Therefore, asciminib should be considered a targeted option for selected patients with T315I-mutated CML, with treatment decisions informed by comprehensive mutational profiling and disease context.

Within a mutation-guided therapeutic framework, BCR-ABL1 KD mutation analysis is recommended in patients with suspected treatment failure, as it informs rational selection of subsequent TKIs according to mutation-specific sensitivity profiles [30,154,155,156]. In cases of advanced-phase disease or high-risk resistance, management commonly incorporates intensive combination chemotherapy, with or without a TKI, as a bridge to allo-HSCT when feasible [157,158].

Although BCR-ABL1 KD mutations represent a well-established mechanism of resistance in patients with CML who relapse during TKI therapy, resistance may also occur in the absence of detectable mutations. In such cases, resistance is thought to arise from BCR-ABL1-independent mechanisms, including persistent or reactivated signaling through downstream pathways such as MAPK, PI3K/AKT, SRC family kinases, and JAK/STAT, which can sustain LCS despite effective BCR-ABL1 inhibition [146,147].

Kinase-independent resistance in CML is frequently associated with additional genetic alterations, although the precise mechanisms remain incompletely understood. In blast crisis, multiple coexisting genetic abnormalities have been described, including point mutations in IKZF1, RUNX1, ASXL1, BCORL1, and IDH1/2; gene fusions involving MLL, MSI2, and MECOM; and deletions affecting PAX5, CDKN2A, HBS1L-MYB, and 17p [159,160,161]. Additional chromosomal abnormalities, such as trisomy 8, trisomy 19, and isochromosome 17q, are frequently acquired during progression to blast crisis in CML and contribute to the complex genomic landscape of advanced-phase disease [162,163,164].

Among non-BCR-ABL1 lesions, ASXL1 mutations have the most consistent prognostic significance in chronic-phase CML. ASXL1 mutations detected at diagnosis are associated with adverse disease biology, including higher-risk clinical features and inferior outcomes, reflecting early clonal complexity in CML [165,166]. These mutations have consistent prognostic value, correlating with reduced response durability and survival. However, ASXL1 mutations have not been shown to reliably predict response to any specific TKI and are therefore considered prognostic rather than predictive biomarkers [165,167]. Accordingly, ASXL1 mutations contribute to risk stratification and long-term disease monitoring rather than informing frontline TKI selection. A similar pattern is observed with other non-BCR-ABL1 lesions, whose prognostic relevance and potential role in guiding therapeutic decisions in chronic-phase CML remain incompletely characterized.

Overall, TKI resistance in CML is driven by both BCR-ABL1-dependent and -independent mechanisms, compounded by additional genetic and chromosomal alterations. Integrating comprehensive mutational and cytogenetic profiling with clinical risk assessment would assist in guiding personalized therapy, optimizing TKI sequencing, and identifying patients who may benefit from advanced interventions or early allo-HSCT.

## 7. Managing Resistance and Discontinuation in Chronic Myeloid Leukemia

Therapeutic sequencing in CML after TKI for intolerance or resistance adheres to evidence-based principles prioritizing rapid switch to alternative agents, guided by mutation profiling, comorbidity assessment, and patient preferences [168,169]. TKI intolerance, often presenting as persistent low-grade toxicities that interfere with daily activities, is managed through dose optimization or switching to better tolerated TKIs [12,170]. According to the 2025 ELN recommendations, treatment resistance is characterized by failure to achieve key molecular response milestones, defined as BCR-ABL1 > 10% at 3 or 6 months, >1% at 12 months, or a loss of MMR thereafter, and should prompt BCR-ABL1 kinase domain mutation analysis to guide subsequent targeted therapy selection [30].

Several prospective trials validate early, mutation-guided, and intolerance-driven TKI sequencing in chronic-phase CML. The phase II TIDEL-II trial exemplified the merits of a response-directed therapeutic sequencing paradigm, wherein imatinib dose intensification or timely transition to nilotinib was employed for patients not meeting established molecular benchmarks or manifesting treatment-related intolerance. Dose escalation of imatinib salvaged durable responses in only a limited subset of patients, approximately 11%, whereas transition to nilotinib facilitated achievement of MMR in an additional 15% by 24 months, particularly among those undergoing intolerance-mediated switches, which correlated with elevated rates of subsequent efficacy. In aggregate, this sequential strategy resulted in cumulative MMR rates of 73% and MR4.5 rates of 34% at 24 months, highlighting the value of therapeutic sequencing in enhancing sustained outcomes [171]. Post hoc evaluations from the phase III ENESTnd trial indicated that response milestone-directed dose intensification or agent switching in cases of suboptimal efficacy led to CCyR in 58% and MMR in 32% of affected individuals [124]. The prospective NEXT-in-CML investigation employed NGS to identify low-burden kinase domain mutations in 34% of participants exhibiting warning or failure responses, thereby guiding mutation-specific therapeutic adjustments, including ponatinib for T315I variants, and yielding clinically implementable regimen alterations in 18% of cases [58]. In advanced-line scenarios, the phase III ASCEMBL trial established the superiority of asciminib relative to bosutinib among individuals with resistance or intolerance to at least two prior TKIs, demonstrating MMR rates at 24 weeks of 25.5% vs. 13.2%, complemented by a superior safety profile that facilitates comorbidity-tailored sequencing [74].

Management differs markedly between mutation-positive and mutation-negative resistance. Mutation-positive resistance to ATP-competitive TKIs often involves specific point mutations, such as T315I, or compound mutations. In these settings, targeted agents such as ponatinib, the allosteric inhibitor asciminib, or the newer third-generation TKI olverembatinib are preferred, guided by kinase domain mutation profiling [172,173]. Ultra-deep NGS improves sensitivity for detecting low-level emergent mutations, as low as 1–2%, enabling earlier detection and pre-emptive switching compared with conventional Sanger sequencing [174]. Mutation-negative resistance frequently arises through BCR-ABL1-independent mechanisms, including the activation of alternative signaling pathways, such as MAPK/ERK, PI3K/AKT, and JAK/STAT, or the persistence of quiescent LSCs [147,175]. In the absence of kinase domain mutations, therapeutic strategies emphasize combining TKIs with agents targeting parallel survival pathways, including JAK/STAT inhibition with ruxolitinib, PI3K/AKT/mTOR blockade with agents such as copanlisib or everolimus, autophagy inhibition with hydroxychloroquine or Lys05, and BCL-2 targeting with venetoclax [147].

Eligibility for TFR requires strict criteria to minimize the risk of relapse. Patients must be in chronic-phase CML with typical BCR-ABL1 transcripts (e13a2 or e14a2), have received TKI therapy for more than 5 years (≥4 years for 2G-TKI), and demonstrate a sustained DMR, defined as ≥3 years of MR4.0 or ≥2 years of MR4.5 [176,177]. Monitoring during TFR is intensive to detect early molecular relapse. Guidelines recommend qPCR for BCR-ABL1 every month for the first 6 months, bimonthly in within 7–12 months, and every 3 months thereafter, with prompt TKI resumption at MMR loss [30,176,177,178].

Molecular recurrence represents a frequent occurrence following TKI discontinuation in patients attempting TFR, with the majority of events manifesting within the first 12 months [39,77,179,180,181,182,183]. Pooled analyses from earlier clinical trials, which predominantly employed imatinib as first-line therapy, documented molecular recurrence in approximately 41% of patients by 6 months, increasing to 51% over longer follow-up periods [184]. Later analyses that incorporated studies utilizing 2G-TKIs in the frontline setting have reported comparable relapse kinetics, with rates of 34% at 6 months and 41% cumulatively [185]. Late relapses (>36 months) affect approximately 10%, often linked to residual leukemic stem cells [186]. Retreatment outcomes are favorable, with 98% regaining MMR and DMR upon TKI resumption, highlighting TFR’s reversibility [185]. The high rates of molecular relapse reversibility, along with effective mutation-guided sequencing, highlight that CML has evolved into a chronic, precision-managed disease in which long-term outcomes are optimized through adaptive therapy rather than fixed treatment strategies.

## 8. Emerging and Future Therapeutic Strategies

Although current TKIs and molecular monitoring strategies have transformed CML into a largely manageable disease, several unmet needs related to treatment efficacy, tolerability, and decision-making persist [187]. As survival outcomes plateau, future therapeutic strategies are increasingly focused on deeper molecular eradication, targeting LSCs, and refining combination and next-generation approaches to enable safer and more durable remission [188,189].

TKIs, while highly effective, cannot eradicate quiescent LSCs that drive relapse [188,190]. Achieving durable remission requires strategies beyond TKIs, targeting both stem cell survival and immune regulation [190]. Combination therapies are a major focus: IFN-α can sensitize LSCs and enhance immune surveillance, while agents like venetoclax, ruxolitinib, and pioglitazone target apoptosis, survival pathways, and quiescence, respectively [188,190]. Parallel pathway inhibition (PI3K/AKT/mTOR, MAPK, autophagy) may further reduce resistant clones [145,191]. Immunological approaches, including vaccines against BCR-ABL1/WT1 and checkpoint inhibitors, aim to restore anti-leukemia immunity, with natural killer cells playing a key role in sustaining remission [188,190]. Early-phase trials of asciminib combined with TKIs demonstrate rapid and durable molecular responses, supporting combination strategies to expand TFR eligibility [192]. Such approaches may ultimately allow more patients to safely discontinue TKIs while maintaining long-term remission [188,190].

The clinical success of asciminib has validated allosteric inhibition of the BCR-ABL1 myristoyl pocket as a viable therapeutic strategy in CML, providing a mechanistic foundation for the development of next-generation agents beyond first-generation STAMP inhibitors [193,194,195,196]. Preclinical and structural studies demonstrate that allosteric-orthosteric co-binding can enhance kinase suppression by stabilizing inactive BCR-ABL1 conformations and improving ATP-competitive inhibitor affinity [195,196]. Complementary biochemical analyses show that targeting regulatory sites outside the ATP-binding pocket restricts kinase activation dynamics, providing a mechanistic basis for potent and synergistic combinations with ATP-competitive TKIs and improved target specificity [195,196].

Building on this concept, novel allosteric inhibitors such as TERN-701 are now entering clinical evaluation, aiming to improve efficacy and safety in heavily pretreated CML populations, including patients previously exposed to asciminib or ponatinib [197]. Early-phase data from the CARDINAL study show favorable tolerability and durable molecular responses, reinforcing the feasibility of advancing alternative myristoyl pocket inhibitors [197]. Unlike traditional TKIs, asciminib can be combined with ATP-competitive inhibitors such as nilotinib or ponatinib, including in resistant and T315I mutant contexts, to enhance kinase suppression and overcome resistance through complementary allosteric and ATP site inhibition [195,196]. Structural and biochemical data confirm that this dual inhibition is synergistic without steric interference, leading to deeper suppression of BCR-ABL1 signaling and improved target specificity [196]. In contrast, antagonistic interactions with other TKIs highlight the need for rational pairing rather than empiric combination [195].

CML exemplifies precision oncology, driven by the BCR-ABL1 fusion gene, which serves as both a diagnostic biomarker and therapeutic target [37,198,199]. TKIs have transformed outcomes, while molecular monitoring, primarily via quantitative PCR, guides disease assessment, relapse prediction, and treatment discontinuation in patients achieving DMRs [37,198]. Resistance from ABL KD mutations and clonal heterogeneity remains a challenge, motivating advanced monitoring strategies [14,37]. Emerging approaches, including digital PCR, NGS, and single-cell genomics, enable detection of minimal residual disease, low-frequency mutations, and insights into clonal evolution, informing therapy optimization [37,198,199]. Beyond CML, multi-omics profiling and AI-driven analytics are beginning to reshape diagnosis, risk stratification, and treatment strategies across hematologic malignancies [199].

Together, these developments signify a transformative shift in CML management, from uniform TKI therapy towards adaptive, patient-centered strategies that integrate novel targeted agents, combination therapies, and advanced monitoring to achieve durable, safe, and potentially TFR.

## 9. Conclusions

The management of CML has evolved from a survival-focused approach to one centered on long-term disease control, QoL, and TFR. While TKIs have transformed outcomes across all disease phases, the expanding therapeutic landscape has introduced new complexities in treatment selection, monitoring, and survivorship care. Achieving early and sustained DMRs remains fundamental, not only for minimizing disease progression but also for identifying patients eligible for TFR.

Frontline and sequential TKI selection now requires careful integration of molecular efficacy, toxicity profiles, CV risk, patient comorbidities, and individual treatment goals rather than reliance on baseline risk scores alone. 2G-TKIs offer faster and deeper responses but at the cost of distinct long-term toxicities, while asciminib introduces a novel option with promising efficacy and tolerability. Ultimately, optimal CML care demands a personalized, adaptive treatment strategy that aligns durable molecular control with lifelong safety.

However, reliance on conventional RT-qPCR alone is increasingly challenged by technical limitations near deep response thresholds, highlighting the growing importance of more sensitive and reproducible tools such as ddPCR and NGS. Future research priorities should focus on the clinical standardization and harmonization of these high-sensitivity molecular assays to ensure reproducibility and consistent interpretation across centers. Long-term safety and CV outcome data for newer TKIs, particularly in younger patients and those pursuing TFR, remain essential. Integrating evolving molecular tools with patient-reported outcomes and comorbidity-driven TKI selection will be critical for expanding the evidence base into everyday clinical decision-making.

## Figures and Tables

**Table 1:** Cytogenetic and molecular response categories in CML.

Response Type	Definition	Measurement	Clinical Relevance
CCyR	No detectable Ph^+^ metaphases in ≥20 bone marrow metaphases	Bone marrow cytogenetics	Associated with prolonged PFS and reduced risk of progression; historical therapeutic benchmark
MMR, MR3	BCR-ABL1 IS ≤ 0.1% (3-log reduction from baseline)	RT-qPCR	“Safe harbor” response with minimal risk of progression; key long-term treatment goal
DMR	MR4 (≤0.01%), MR4.5 (≤0.0032%), MR5 (≤0.001%)	RT-qPCR, ddPCR	Required for TFR; reflects profound and sustained disease suppression
EMR	BCR-ABL1 IS ≤ 10% at 3 months	RT-qPCR	Early predictor of long-term outcomes, including achievement of MMR/DMR
Loss of Molecular Response	Loss of MMR or ≥1-log increase in BCR-ABL1	RT-qPCR	Requires clinical intervention: adherence assessment, mutation testing, and treatment modification

Abbreviations: CCyR, complete cytogenetic response; MMR, major molecular response; DMR, deep molecular response; EMR, early molecular response; IS, International Scale; RT-qPCR, real-time quantitative PCR; ddPCR, droplet digital PCR.

**Table 2:** Comparative efficacy, toxicity, cardiovascular risk, and treatment-free remission potential of commonly used frontline TKIs in chronic-phase CML.

TKI	Efficacy (CCyR/MMR/DMR)	Key Toxicities	Cardiovascular Risk	TFR Potential	Recommended Patient Profile/Notes
Imatinib	High CCyR; moderate MMR; slower DMR	Edema, muscle cramps, gastrointestinal intolerance	Low: cardiovascular-sparing	Moderate; slower DMR may delay TFR eligibility	Older or frail patients; high baseline CV risk; those prioritizing long-term tolerability
Dasatinib	Faster CCyR and MMR; higher rates of early DMR	Pleural effusions, pulmonary arterial hypertension, cytopenias	Moderate; pulmonary vascular toxicity	High; rapid DMR favors TFR	Patients seeking early deep response; low CV risk; requires pulmonary monitoring
Nilotinib	Faster CCyR and MMR; higher DMR rates vs. imatinib	Hyperglycemia, dyslipidemia, QT prolongation	High; ischemic heart disease, arrhythmias	High; rapid DMR favors TFR	Younger patients with low CV risk; strong TFR intent; requires strict CV and metabolic monitoring
Bosutinib	Faster early MMR; DMR rates comparable to other 2G-TKIs	Diarrhea, hepatotoxicity, cytopenias	Low-to-moderate; less clearly defined	Moderate–high	Patients with CV comorbidities; monitor hepatic function and gastrointestinal tolerance
Ponatinib	High efficacy; active against T315I	Arterial/venous occlusion, hypertension	Very high; major vascular events	Limited; TFR is less studied	Resistant CML, T315I mutation; high CV risk requires caution
Asciminib	Effective after ≥2 prior TKIs; high MMR	Cytopenias, lipase elevation, pancreatitis	Low-to-moderate	Promising; ongoing studies	Multi-resistant patients; allosteric inhibitor spares off-target effects; good tolerability

Abbreviations: TKI, tyrosine kinase inhibitor; CML, chronic myeloid leukemia; CCyR, complete cytogenetic response; MMR, major molecular response; DMR, deep molecular response; TFR, treatment-free remission; CV, cardiovascular; QT, corrected QT interval.

## Data Availability

No patient data were directly utilized in this study.

## Abbreviations

The following abbreviations are used in this manuscript:

CML	Chronic Myeloid Leukemia
IFN-α	Interferon-alpha
Ph	Philadelphia
TKI	Tyrosine Kinase Inhibitor
Allo-HSCT	Allogeneic Stem Cell Transplantation
OS	Overall Survival
DMR	Deep Molecular Response
TFR	Treatment-Free Remission
QoL	Quality of Life
CV	Cardiovascular
CCyR	Complete Cytogenetic Response
RT-qPCR	Real-Time Quantitative Polymerase Chain Reaction
IS	International Scale
IRIS	International Randomized Study of Interferon and STI571
MMR	Major Molecular Response
MR4	Molecular Response with a 4-log Reduction
MR4.5	Molecular Response with a 4.5-log Reduction
MR5	Molecular Response with a 5-log Reduction
ELN	European LeukemiaNet
ACA	Additional Chromosomal Abnormality
ddPCR	Digital Droplet Polymerase Chain Reaction
NGS	Next-Generation Sequencing
LSCs	Leukemic Stem Cells
CVD	Cardiovascular Death
KD	Kinase Domain
